# Relationship Between Health Benefit Perception Moderate Wine Consumption, Wine Label and Healthy Behaviour

**DOI:** 10.3390/foods14111937

**Published:** 2025-05-29

**Authors:** Anita Silvana Ilak Peršurić, Ana Težak Damijanić, Sanja Radeka

**Affiliations:** 1 Department Economics and Agricultural Development, Institute of Agriculture and Tourism, 52440 Poreč, Croatia; anita@iptpo.hr; 2 Department Tourism, Institute of Agriculture and Tourism, 52440 Poreč, Croatia; 3 Department Agriculture and Nutrition, Institute of Agriculture and Tourism, 52440 Poreč, Croatia

**Keywords:** consumers, wine, wine label, food, health benefits, Croatia

## Abstract

Moderate wine consumption is, generally, the focus of various medical studies, while consumer behaviour research does not specifically centres on moderation in wine consumption. Wine consumption in moderation is an important part of various healthy diets; still, consumers need to make informed choices when purchasing wine and the information printed on wine labels partially contributes to this process. Therefore, the main aims of this paper were to develop a scale for measuring perceptions of the health benefits associated with moderate wine consumption, and to test the effect of dietary habits and non-obligatory wine label information on the perception of the health benefits associated with moderate wine consumption. The data were collected on a sample of wine consumers who participated in an interdisciplinary experiment regarding the impact of moderate wine consumption on human health. Univariate, bivariate, and multivariate statistics were used. The consumers’ socio-demographic characteristics were used as a starting point in the analysis because they influence wine consumption. Gender was identified as a consistently important variable in predicting the perception of health benefits associated with moderate wine consumption. Health behaviour was a significant predictor along with gender, but after introducing non-obligatory wine label information, its significance in explaining the dependent variable was diminished. The results suggest that a consumer’s perception of the scale of moderate wine consumption is a unidimensional construct. Furthermore, the non-obligatory information on wine labels was identified and classified as either wine-related warnings or wine-related health benefits.

## 1. Introduction

Wine consumption has been an important part of different healthy diets [1,2,3], like the Mediterranean diet, for centuries [4], and it has been a particularly popular choice of beverage with a meal [5,6]. However, since wine is an alcoholic beverage and recent health-related studies have stressed both its negative and positive impacts on consumers’ health, its consumption may be regarded with caution [7,8]. Nevertheless, moderate wine consumption is still considered a crucial part of the Mediterranean diet and other diets considered healthy [6,9,10,11,12], with the emphasis on the word “moderate”. Although the literature does not strictly define what moderate wine consumption means [7,13,14], it generally means limiting wine consumption to a maximum of 300 mL per day. Moderate wine consumption may help in dealing with different diseases [13,15,16,17,18,19,20]. These benefits could prompt consumers to consume wine, and the information that wine producers put on wine labels may help them in their purchasing process. However, consumers are often confused about the positive influence that moderate wine consumption can have on their health [7]. Therefore, appropriate information should be provided for them, and this information should include information about the health benefits of wine. Furthermore, for consumers to make an informed choice, they also need to know the potential side effects, which may be considered to be wine-related warnings.

Current research regarding moderate wine consumption generally focuses on various medical studies [13,15,16,17,18,19,20], while research regarding healthy diets mostly mentions that moderate wine consumption is a part of a particular diet [9,10,11,12]. Furthermore, non-obligatory wine label information is rarely investigated [21,22,23,24]. Therefore, there is a lack of research focusing on how consumers perceive the health benefits of moderate wine consumption, especially in consumer research. Consequently, there is also a lack of research in the consumer research field about moderate wine consumption and healthy diets, and moderate wine consumption and non-obligatory wine label information. Therefore, the aims of this paper were to develop scales for measuring the perception of health benefits associated with moderate wine consumption and non-obligatory wine label information, and to test the effect of dietary habits and non-obligatory wine label information on the perception of health benefits associated with moderate wine consumption.

## 2. Literature Review

### 2.1. Moderate Wine Consumption and Health Benefits

Wine consumption is often a part of the human diet, especially in traditionally wine-producing countries [1,2,3], and it is usually consumed as a part of a meal [6]. However, excess alcohol intake has a negative impact on human health [13,22,23,24]; therefore, wine should be consumed in limited quantities, because moderate wine consumption is generally perceived as beneficial to one’s health [23,25]. However, there are slight differences in descriptions of what moderate wine consumption means.

Moderate wine consumption, according to [13], refers to the intake of less than 15.0 g of alcohol per day, which is equivalent to about one drink. Contrary to their methodology, [7] accept the definition of moderate wine consumption as the consumption of 200 mL of wine for women and 300 mL of wine for men, which equates to around an average of 20 g or 30 g of pure alcohol, respectively. The latter definition might be considered a kind of compromise between the strict limitation provided by [13] and the findings of [7] that have determined that wine consumers generally consider one glass of wine moderate, but 38% of Italian wine consumers and 22% of Spanish wine consumers believed that two to three glasses are still moderate. This definition of “moderate” wine consumption is also supported by [14] who identified that 40% of Italian, 20% of Spanish, and 12% of French participants considered two glasses a day to be moderate wine consumption.

Although there are still some disagreements about what moderate wine consumption includes, health research focusing on wine’s benefits has identified its different positive effects on human health [13,15,16,17,18,19,20]. Those effects are related to cognitive, metabolic, and cardiovascular diseases. Both men and women drinking light to moderate amounts of wine daily scored better on all cognitive tests compared to those respondents drinking other alcoholic beverages [15]. Moderate wine drinkers, especially women, demonstrated better cognitive scores compared to non-drinkers [13] and male wine drinkers had reduced a risk of Alzheimer’s disease [16,26]. Additionally, medical research has identified the benefits of moderate wine consumption regarding different neurological problems [17,22,23,24], especially in protecting neuronal cells from damage, preventing neurodegenerative diseases, facilitating the treatment of depression, improving cognitive function, and increasing memory [27]. One study, conducted during the COVID-19 pandemic, based on the research performed by [28], identified that the anti-microbe and anti-bacterial properties of certain wine components have a positive impact on COVID-19-related health issues [29]. Furthermore, the positive effects of wine on cardiovascular diseases were confirmed due to the presence of polyphenols, mainly resveratrol, in wine [18]. Finally, some authors reported that moderate wine consumption may grant people a longer life expectancy, compared to consuming wine in excess or not consuming wine at all [19,20].

Various medical studies, to a certain degree, influence public opinion about wine consumption. Positive aspects are usually the basis for a perception of health benefits. The health benefits that consumers generally link to moderate wine consumption include different aspects such as lowering cholesterol levels and reducing the risk of cardiovascular diseases [7,30]. Furthermore, wine consumers believe that wine has a positive effect on atherosclerosis, hypertension, cancer, type 2 diabetes, and neurological disorders [8]. However, wine consumers had a slightly negative perception of how wine influences sleep [7].

#### 2.1.1. Wine Consumption and Diet

The moderate consumption of wine is often a part of a healthy diet; however, the main question is what is a healthy diet. Different sources stress diets like the Mediterranean, DASH, MIND, and traditional Asian diets as healthy diets [9,10,11,12]. Some of them are characteristic of certain regions like the Mediterranean region and are well known worldwide. The primary prerequisites of all healthy diets are variety in the intake of food and the limitation of the quantities consumed. This often results in the minimisation of fats, sugar, processed and readymade foods, etc., and the encouragement of legume intake, fruit and vegetable intake, etc., in order to improve one’s health [31,32,33,34]. Consumers practising one of these healthy diets should experience different health-related improvements by diminishing the risks related to obesity, high blood cholesterol, coronary diseases, and type two diabetes [31,35]. Healthy diets often include moderate wine consumption because moderate wine consumption contributes by a certain degree to the achievement of different health benefits [7,30]. For instance, when wine is a part of a healthy diet, it is consumed as a part of a meal [4,6,36], so its negative effects on human health are diminished [37].

#### 2.1.2. Moderate Wine Consumption and Label Information

Consumers’ food purchase-related choices are often based on information provided on product labels. Information on product labels is not unified worldwide [38]; however, all product labels must contain information that is mandatory, as defined by different countries [14,38]. The information presented on wine labels has a certain impact on consumers’ intention to purchase and consume wine [30,39]. Wine labels often include information about the wine’s extrinsic characteristics, i.e., the grape variety, region, country, vintage [40], and this information could be regarded as obligatory information; however, other aspects related to wine, like warnings about the harmful consequences of wine consumption [23] or stating the health benefits that moderate wine consumption can have on consumers health [25], are generally not mentioned as information on wine labels. Furthermore, there is no agreement worldwide about the information that should be stated on wine labels [23,41]. Nevertheless, the information presented on wine labels is important to consumers, although its importance and content might vary due to different consumer characteristics, like their socio-demographic characteristics [23,41,42]. However, nowadays consumers might have a problem understanding what is written on the label. Some authors [43] identified that consumers were confused about food labels in general; namely, they often had difficulties identifying whether some label content (especially nutritional components) is important or valuable to the overall population, but they had even more problems with food labels containing health claims.

#### 2.1.3. Wine Consumption and Socio-Demographic Characteristics

In general, consumers’ socio-demographic characteristics influence their food purchasing decision-making process, and the same applies to wine consumption [8,14,39,44]. The socio-demographic characteristics of consumers were identified as important in relation to their nutrition [14], their perception of wine as a product [25], wine label information [22,23,24,39], and wine consumption habits [45]. Regarding wine consumption, the sociodemographic characteristics of consumers are generally used as segmentation criteria [14,44,46]. Wine-related research has identified gender [8,14,24,25,41,44,45,47], age [8,23,25,30,39,42,45,48,49], education [8,24], occupation [8], and income [8,45] as the most relevant consumer characteristics. Considering consumers’ gender, [23] determined that women are more interested in information on wine labels compared to men, while [25] identified that women are more likely to believe that sulphites in wine may give some people headaches and that men were prone to believing that white and rosé are healthier wine types. Age was also identified as an important demographic characteristic in relation to wine consumption; namely, [39] determined that Generation X reported higher levels of subjective wine knowledge compared to Generation Y, while the findings of [25] suggest that millennials were most likely to consider white, rosé, and sparkling wines as healthier wine types compared to the other age groups. Furthermore, [30] determined that those wine consumers who identified a few wine-related health benefits tended to be older. Income was a significant variable in predicting consumers’ spending on wine [45], while occupation and income were differentiating factors among wine consumer segments identified by [8].

In summary, consumers’ socio-demographic characteristics, their health behaviour, and the information on wine labels can influence consumers’ wine consumption; therefore, this paper poses the following hypotheses:

 **H1:**  *Gender has a positive influence on moderate wine consumption.*

 **H2:**  *Age has a positive influence on moderate wine consumption.*

 **H3:**  *Education has a positive influence on moderate wine consumption.*

 **H4:**  *Income has a positive influence on moderate wine consumption.*

 **H5:**  *Profession has a positive influence on moderate wine consumption.*

 **H6:**  *Health behaviour has a positive influence on moderate wine consumption.*

 **H7:**  *Wine warnings have a negative influence on moderate wine consumption.*

 **H8:**  *The perception of wine-related health benefits has a positive influence on moderate wine consumption.*

## 3. Materials and Methods

### 3.1. Research Design

The research presented in this paper is part of a wider research project conducted as a part of the Vinum Sanum project financed by the Croatian Science Foundation (HRZZ), aiming to examine moderate wine consumption in relation to human health. The Vinum Sanum project is a multidisciplinary scientific project that includes experts from the life sciences (agronomy and medicine) and social sciences and humanities (economy and psychology). Therefore, in the sample design process, the main characteristics related to both research fields were considered. The data collected from participants included variables regarding the participants’ health (medicine), the results of a wine consumption experiment (agronomy), and their consumer behaviour (economy and psychology). The research was conducted in Istria County and Primorsko-goranska County in Croatia, and it included the local population. Data were collected during 2019 and 2020 by researchers. For each year, a minimum sample size of 200 participants was planned.

The selection of participants was influenced by medical and agronomy research requirements; namely, the following categories were excluded: people with physical and mental health problems, people with addiction problems, and pregnant and lactating women. Furthermore, the participants’ health-related variables were collected before and after the application of the wine consumption experiment, and no interventions regarding the participants’ diet or physical activity were carried out. The participants were recruited based on public announcements published in different printed and online media. They were asked to submit their application, and the first 200 applicants satisfying the previously mentioned requirements were selected. The researchers invited the selected participants, and the following tests were performed before they were accepted as research participants: a routine medical exam, blood tests, and an interview with a psychologist. Only healthy individuals were included in the research and placed in a control group or wine consumer group. They all signed a letter of consent after being informed about the research. The Ethical Committee approved this research. The participants consumed 200 mL of wine daily during mealtimes for six weeks. All participants were asked to maintain their usual dietary habits during the consumption period, and to abstain from other alcoholic beverages except for wines provided by the project team. During the medical part of the project, medical examinations and laboratory tests were conducted for all study participants. The medical examination included measuring the participant’s weight, height, and waistline, and their hip width and BMI. Heart rate and systolic and diastolic blood pressure were measured, and an electrocardiogram (ECG) was obtained. Data regarding health, therapy, smoking, and alcohol consumption habits were recorded. Laboratory tests included routine biochemistry and haematology analysis and the determination of serotonin and dopamine concentrations.

### 3.2. Consumer Behaviour Research

The data presented in this paper were collected as part of consumer behaviour research in the field of economics, and they included only participants who regularly drank moderate amounts of wine and were older than 18 years of age, namely the wine consumer group. The participants were asked to fill in the questionnaire only once; that is, at the same as when the researchers conducted the previously mentioned medical exams and the interview with a psychologist.

The questionnaire containing 23 questions was divided into three sections—wine consumption-related questions (wine consumption motivation, wine consumption perception, wine-related information, and wine attributes), dietary habits, and the participants’ socio-demographic characteristics. All the scales used in this research were constructed based on previous research (see Appendix A).

The moderate wine consumption scale measured the consumers’ perception of the benefits that limited wine consumption has for their health. It was operationalized as a unidimensional construct and the items for measuring the health benefits of moderate wine consumption were generated based on research proposed by [7,12,13,15,16,50,51]. Initially, 10 items were generated. A Likert-type scale ranging from 1 to 5 (1—Not important at all, 2—Not important, 3—Neutral, 4—Important, 5—Very important) was used. Dietary habits were measured as a unidimensional construct, and the items were adopted from [10,12,51,52,53,54,55]. To measure dietary habits, a total of 17 items were used. A Likert-type scale ranging from 1 (totally not agree) to 5 (totally agree) was used. Questions about non-obligatory wine label information were drawn from [7,8,22,23,24,56,57,58], and they were measured using a scale from 1 to 5 (1—not important, 2—minimally important, 3—medium importance, 4—very important, 5—exceptionally important). This construct was operationalised as a two-dimensional construct; namely, four items measuring wine’s health benefits and four items measuring wine-related warnings were generated. All constructs were first examined by a psychologist and then by three experts (agronomy, medicine, and economy) to achieve content adequacy, and later they were tested on 10 volunteers working at the Institute of Agriculture and Tourism, who were not directly related to the project, to examine the clarity of each statement [59].

### 3.3. Data Analysis

Data were processed using IBM SPSS software version 21 and included descriptive statistics (to provide a general description of the sample), bivariate statistics (correlation), and multivariate statistics (exploratory factor analysis to determine the dimensions of the constructs and regression analysis to test the relationships between the perception of health benefits associated with moderate wine consumption and dietary habits and non-obligatory wine label information). Prior to data processing, the individual items measuring the health benefits of moderate wine consumption, wine-related information, and dietary habits were examined by checking data accuracy, missing data, and distribution. Missing values were replaced using the Markov chain Monte Carlo (MCMC) method for item imputation. To identify potential dimensions or to determine the unidimensionality of constructs, exploratory factor analysis was applied, using the maximum likelihood method and Promax rotation with an eigenvalue of 1.00 or more to identify potential factors. Internal reliability was determined by computing Cronbach’s alpha. The factors of the perception of health benefits associated with moderate wine consumption, non-obligatory wine label information, and dietary habits were calculated as a mean value for each respondent [60,61,62]. To test the influence of dietary habits and non-obligatory wine label information, a hierarchical regression was conducted. Correlation between independent variables was calculated prior to the performance of hierarchical regression. Hierarchical regression was chosen to examine the effect of dietary habits and non-obligatory wine label information on the perception of health benefits associated with moderate wine consumption [63]. Since socio-demographic characteristics were identified as factors influencing the wine consumers’ behaviour, they were used as control variables. Dietary habits were introduced in the second step to determine the proportion of variance explained by the respondent’s diet, and then non-obligatory wine label information variables were introduced to test their influence on the perception of health benefits associated with moderate wine consumption. To test for multicollinearity, the variance inflation factors (VIFs) were calculated.

## 4. Results

### 4.1. Sample Characteristics

The sample consisted of 357 moderate wine consumers identified as adequate to participate in the research. Regarding the participants’ gender, the proportion of the female respondents was higher than that of male respondents (Table 1). The majority of the participants were 41 years of age or older. Based on their education, the participants were divided into two segments, elementary/secondary and tertiary (since less than 0.7% had only elementary education). The participants were divided into either an employee category or others (this category included students (7%), retirees (5%), and those not in employment (4.6%)). Income was measured in the former Croatian currency (kuna) and was later calculated in euros (therefore, the range is not a round number). The majority of the respondents had a personal monthly income below EUR 929.2.

The relationships between wine consumption and food and diets (Table 2.) were evaluated by participants with five grades that variated from the most negative (not at all) to the most positive (very important). About ¾ of the survey participants consumed wines mostly at home. Our findings on consumption frequency were similar to the pattern found in Spain by [44], where the home consumption pattern was the most frequent one (places of consumption: at home, restaurants, bars, and other places). The preferred wine type for the majority of participants was dry wines. A minority of participants preferred special wine types, e.g., sparkling and liqueur wines. Considering the frequency of wine consumption, every fifth participant consumed wine daily and one out of three consumed wine several times a week.

Wine was evaluated in a positive sense (Table 3), as a beverage with positive health aspects; namely, as good for one’s health, as a natural beverage, and as part of a healthy diet. In over sixty percent of cases, this evaluation was important or very important. In the evaluation of their diet (Table 3), the participants expressed their serious concerns about their food choices and particular food components. Their awareness about the influence of food on their health was evident and taking care of their own health was validated as highly important (six out of ten participants found it was important or very important). Consequently, seven out of ten participants evaluated the impacts of food on their health as very important. Their food choices revealed preferences for fresh food in their daily diet, consequently fresh food consumption was very important or important to three-quarters of the participants. In accordance with their fresh food choices, their cooking practices also revealed a healthy preference for cooking at home from scratch. Consequently, less than ten percent of participants regularly consumed prepared/readymade meals or opted for fast food in their diet.

Regarding particular compounds in food, such as the content of fats, sugar, salt, vitamins, and additives in food, the participants were divided, with half of them stating that these food compounds should be counted and controlled, about one-third finding this to be neither important nor unimportant, and the rest finding it to be of low or no importance.

The evaluation of diet, food components, and wine as part of a daily diet showed that the survey participants were highly aware of food, beverages, and their impact on health. The encompassed data also indicate that the sample was divided into the half who evaluated all statements highly, about a third who were indecisive, and the ones who found it to be of low importance. Consequently, it was presumed that certain socio-demographic features of the participants may have affected this division of the sample.

### 4.2. Scale Analysis

Exploratory factor analysis was performed to identify the dimensions of the perception of health benefits associated with moderate wine consumption and dietary habits and non-obligatory wine label information (Table 4). Items with a loading below 0.5 and cross-loadings were deleted, resulting in the retention of 26 items—nine items measuring the health benefits of moderate wine consumption (a unidimensional construct), eight items measuring dietary habits (a unidimensional construct), and nine items on non-obligatory wine label information (a two-dimensional construct—five items measuring wine-related warnings and five items measuring health benefit claims).

The first factor, F1, measured the perception of health benefits associated with moderate wine consumption. It included different questions regarding how moderate wine consumption can have a positive impact on human health. The items on the perception of health benefits associated with moderate wine consumption varied from moderate consumption having a positive influence on weight (2.9) to moderate consumption having a positive influence on blood vessels (3.7). The second factor, F2, measured dietary habits, and showed an active approach to food choices in daily diet, concerning both the harmful effects of excessive fats (3.1), salt (3.2), sugar (3.4), and additives (3.7), and the possible positive effects of vitamins (3.3) and fibre (3.1). Non-obligatory wine label information was divided into two dimensions. The third factor F3, included items that emphasised the positive impacts of wine consumption on human health, and it was labelled as health benefit claims, while the second factor, F4, contained items related to warnings, and was labelled as wine warnings. Survey participants evaluated the health benefit claims as generally important; namely, they varied from wine contains antioxidative components (3.8) to wine reduces vascular diseases and lowers the risk of heart disease (3.5). However, respondents found wine warnings to be slightly more important; specifically, they rated the warning Do not drink and drive (4.4) as the most important, while Consume in moderate amounts (4.1) was the least important warning. Jointly, all factors accounted for 64.12% of the accumulated variance, and most of the factor loadings were greater than 0.60. Cronbach’s alpha coefficients were between 0.850 and 0.952. The KMO of the data was 0.897, while the significance level of Bartlett’s test was 0.000, which means the data were suitable for factor analysis. All of the items in the scales fall into corresponding factors. Cronbach’s alpha coefficients were above 0.6, which showed the great reliability of all of the scales. Most of the factor loadings exceeded 0.6, suggesting that all of the scales had a high convergent validity. The correlation between an item and the total score (Table 5) was acceptable for all four scales [64].

### 4.3. Model Analysis

To test the effect of dietary habits and non-obligatory wine label information on the perception of health benefits associated with moderate wine consumption, hierarchical regression analysis was performed. Prior to this analysis, descriptive statistics for all four constructs were generated and Pearson’s correlation coefficients were calculated to check if the independent variables were highly correlated. Non-obligatory wine label warnings were the most important aspect (Table 6), but they were slightly more important than non-obligatory wine label health benefit claims. Additionally, the perception of health benefits associated with moderate wine consumption and dietary habits were also important, but they were less important to consumers than non-obligatory wine label information. All constructs used as independent variables were statistically significantly related, but the correlation was relatively low.

Hierarchical regression analysis was used to test the research hypothesis (Table 7). Variance inflation factors varied across the models, but they were between 1.033 and 1.315. Model 1 included control variables only; namely, consumers’ socio-demographic characteristics that explained 4.5% of the variance of the perception of health benefits associated with moderate wine consumption. Gender was the only variable significant in predicting the perception of health benefits associated with moderate wine consumption, and the model was significant at the 0.01 level of significance. In the next step, health behaviour was included.

Although the F test was significant, and health behaviour was also a significant variable, besides gender, the proportion of explained variance increased by 3.5%, increasing R^2^ to only 8%. However, adding health behaviour into this relationship increased the overall level of model significance. In the next step, non-obligatory wine label information was entered; namely, wine warnings and health benefit claims. These two factors increased the proportion of explained variance by 34.8%, increasing R^2^ to 42.8%. After adding these two factors to the regression model, respondents’ gender remained a significant predictor of the perception of health benefits associated with moderate wine consumption. However, health behaviour lost its significance in explaining the dependent variable.

Gender had a negative impact on the perception of health benefits associated with moderate wine consumption, suggesting that women were likely to perceive wine consumption, even if moderate, as not beneficial to human health. Since gender was identified as an important variable in relation to moderate wine consumption, its relationship with the independent variables was tested. Pearson’s correlation coefficients indicated no statistically significant relationships between gender and health behaviour (0.096, sig. 0.077), label warnings (0.080, sig. 0.136), and label health benefits claims (−0.064, sig. 239). Health behaviour had a positive impact on the perception of health benefits associated with moderate wine consumption, suggesting that respondents who pay attention to their diet are more likely to perceive moderate wine consumption to be beneficial to human health. Despite the initial impact of health behaviour in model 2, after adding non-obligatory wine label information, health behaviour lost its significance. Both additional factors were significant; however, wine warnings had a negative impact on the perception of health benefits associated with moderate wine consumption, while health benefit claims had a positive impact on the perception of health benefits associated with moderate wine consumption.

## 5. Discussion

This paper explored wine the relationship between wine consumers’ perceptions of moderate wine consumption, and its relationship to consumers’ dietary habits and the importance they placed on non-obligatory wine label information. First, it assesses the applicability of four measurement scales (moderate wine consumption, health behaviour, wine warnings, and wine health benefit claims). Second, it empirically tests the impact of three different constructs of moderate wine consumption.

All ten items measuring the consumers’ perception of moderate wine consumption loaded significantly onto the factor measuring moderate wine consumption, supporting the findings of [7,12,13,15,16,50,51]. Therefore, this study suggests the scale for measuring consumers’ perception of the benefits of moderate wine consumption includes a comprehensive number of aspects related to the positive influence of wine on human health. Furthermore, the items measuring health behaviour and non-obligatory wine label information loaded significantly onto their factors, confirming the finding related to health behaviour [10,12,51,52,53,54,65] and non-obligatory wine label information [7,8,22,23,24,56,57,58].

By testing the effects of socio-demographic characteristics in relation to moderate wine consumption, this study revealed a strong influence of gender on perceptions of moderate wine consumption, confirming the findings of [8,14,24,25,41,44,45,47]. Namely, men had better opinions about the health benefits of moderate wine consumption compared to women. Health behaviour was a significant predictor along with gender; however, after introducing non-obligatory wine label information, its significance was diminished. These findings suggest that moderate wine consumption is considered an important part of healthy diets, supporting the findings of [9,10,11,12]. However, when non-obligatory wine label information was added, health behaviour was no longer a significant predictor of moderate wine consumption. Both factors representing non-obligatory wine label information were significant in terms of predicting moderate wine consumption, suggesting that non-obligatory wine label information is crucial in relation to consumers’ perception of moderate wine consumption. These results may point to the fact that this information is used in the process forming an opinion on how wine influences human health. Therefore, communicating information on the package or label, especially any health benefits, was found to be important in relation to moderate wine consumption, confirming the findings of [22,23,24,25]. Contrary to the findings of [43], wine consumers were generally well informed about non-obligatory wine label information, recognising both positive and negative wine effects.

## 6. Conclusions

Moderate wine consumption is a part of various healthy diets, but wine consumers need to make informed choices and non-obligatory information on wine labels may help in this process. This paper explored the relationship between wine consumers’ perception of moderate wine consumption, health benefits, socio-demographic characteristics, health behaviour, and wine labels. Wine has various health benefits, but only if consumed in moderation. Consumers in this study recognised the health benefits of moderate wine consumption and generally expressed their healthy behaviour. They also considered both non-obligatory wine label elements, namely health benefit claims and health warnings, to be important, although they placed a higher importance on health warnings. Gender played an important part in relation to consumers’ perception of moderate wine consumption. On the other hand, healthy diet behaviour was an important factor when examined in relation to socio-demographic characteristics. However, when the non-obligatory wine label information variables were added to the equation, health behaviour lost its significance, suggesting that wine-related information plays a greater role in wine consumption compared to diet habits.

This study makes several contributions to the extant literature.

All ten items measuring the consumers’ perception of moderate wine consumption loaded significantly onto the factor measuring moderate wine consumption, supporting the findings of [7,12,13,15,16,50,51]. Therefore, this study suggests a scale for measuring consumers’ perception of the health benefits associated with moderate wine consumption that includes a comprehensive number of aspects related to the positive influence of wine on human health. Furthermore, the items measuring health behaviour and non-obligatory wine label information loaded significantly onto their factors, confirming the finding related to health behaviour [10,12,51,52,53,54,65] and non-obligatory wine label information [7,8,22,23,24,56,57,58].

First, by considering the research proposed by [7,12,13,15,16,50,51], this paper proposes a scale for measuring consumers’ perception of moderate wine consumption. Secondly, by considering the non-obligatory wine label information suggested by [7,8,22,23,24,56,57,58], a scale for measuring wine warnings and wine health benefits was developed. Thirdly, dietary habits adopted from [10,12,51,52,53,54,65] were tested and items measuring them were identified as unidimensional constructs. Finally, the influence of socio-demographic characteristics, dietary habits, and non-obligatory wine label information on moderate wine consumption were evaluated. The findings suggest that gender is a vital predictor of moderate wine consumption. Furthermore, dietary habits exhibit marginal importance in relation to moderate wine consumption, while non-obligatory wine label information plays a major role in predicting perceptions of the health benefits of moderate wine consumption.

This study’s findings bring forth some implications for policymakers and wine producers. Moderate wine consumption is often a part of different diets generally considered to be healthy diets, like the Mediterranean diet. However, consumers must be better informed about what moderate consumption includes in terms of the daily recommended quantity. Therefore, promoting moderate wine consumption as a part of a healthy diet and its benefits on human health is recommended. Although participants in this research were well aware of the health benefit claims and wine-related warnings that can be a part of wine labels, the general population lacks knowledge in this area. Therefore, additional effort should be placed on raising the awareness of the general population in terms of the health benefits of moderate wine consumption, emphasising the word “moderate”. At the same time, they should also be informed about the negative aspects of excessive wine consumption. Non-obligatory wine label information often includes the positive and negative effects of wine on human health. The positive effects of moderate wine consumption could be used to promote different types of functional wines, i.e., wines with increased levels of bioactive compounds. These functional wines could address issues like lowering certain health risks by emphasising different compounds present in wines, like various bioactive compounds with high antioxidative activity. For wine producers, the production of new types of functional wines with additional value enables the diversification of products which enhances their economic status and enforces their long-term competitiveness on the wine market. Raising awareness of the effects of healthy choices of foods and beverages that provide good health, diminish health risks, and prevent diseases is becoming increasingly significant for the general public and also in a scientific sense.

There are a few limitations to this study. The sample consisted of only healthy participants, i.e., neither had any cardiovascular or neurovegetative disease. Future research could include participants with various health issues. The participants were recruited through public announcements published in different media; therefore, the results cannot be generalised to all wine consumers. Future research could test the scales proposed in this study (moderate wine consumption, dietary habits, and non-obligatory wine label information) on wine consumers. This research examined the influence of dietary habits and non-obligatory wine label information on the health benefits of moderate wine consumption. Dietary habits were identified as an important factor in relation to the health benefits of moderate wine consumption. However, they lost their importance when non-obligatory wine label information was added. Since the dietary habits of the participants were not modified for this study, future research could examine in detail the role of dietary habits in general, along with wine’s health benefits and wine-related warnings. Due to the sample design limitations imposed by the multidisciplinary nature of the project, the scales for measuring moderate wine consumption and non-obligatory wine label information were not verified through confirmatory factor analysis. Future research should focus on fixing this issue by testing them on bigger and if possible cross-national samples. Furthermore, future research can centre on applying the findings presented in this paper by extending various consumer behaviour-related theories.

## Figures and Tables

**Table 1:** Socio-demographic features of participants.

N = 357				
Gender	Age (Years)	Education	Occupation	Monthly Personal Income
Female (61.7%) Male (38.3%)	≤40 (42.2%) ≥41 (57.8%)	Elementary and Secondary (25.6%) University (74.4%)	Employee (83.4%) Other (16.6%)	≤€929.1 (54.1%) ≥€929.2 (45.9%)

**Table 2:** Participants’ wine consumption characteristics.

Wine Consumption					
Preferred wine type	Dry 56.8%	Semi-dry 22.8%	Semi-sweet 17.2%	Sparkling 2.4%	Liquor 0.8%
Consumption place	At home 74.2%	Restaurant 21.8%	While travelling 0.3%	In bars, nightclubs 3.5%	Other places 0.2%
Consumption frequency	Special Occasions 16.3%	Several times a year 4.1%	Several times a month 25.5%	Several times a week 32.2%	Daily 21.9%

**Table 3:** Participants’ evaluation of food, food compounds, and wine as part of their diet.

Diet/Food Evaluation	I Do Not Agree at All (%)	I Do Not Agree (%)	I’m Neutral (%)	I Agree (%)	I Totally Agree (%)
Wine is a natural beverage	2.8	2.8	18.6%	40.0	35.8
Wine is good for one’s health	1.9	4.4	23.3%	41.9	28.3
I consume wine as part of healthy diet	6.9	9.4	24.0	37.7	21.9
Wine has positive impact on health	2.8	7.5	23.6	40.3	25.8
I think food affects health	0.6	2.5	5.8	20.6	70.6
I take care of my health	3.1	7.8	28.3	44.5	16.2
I take care of daily calories intake in my diet	11.7	22.6	31.8	26.5	7.3
I take care of daily fat content in my diet	7.8	20.1	32.6	28.7	10.9
I take care of daily sugar content in my diet	6.1	14.8	30.4	33.0	15.6
I take care of daily salt intake in my diet	5.9	18.5	33.6	28.6	13.4
I take care of daily fibre content in my diet	5.6	16.9	40.7	27.5	9.3
I take care of daily vitamin content in my diet	5.9	13.8	34.0	33.1	13.2
I take care of daily additives content in my diet	3.9	10.1	27.0	30.6	28.4
I eat 3–5 meals a day	7.8	12.6	24.1	24.9	30.5
I eat whatever suits me in my diet	18.6	27.8	31.4	13.6	8.3
I prefer fresh food in my diet	2.8	2.8	18.6	40.0	35.8
I prefer to cook or myself	4.4	8.9	12.8	27.2	46.7
I eat often fast-food	58.3	25.3	9.7	5.6	1.1
I prefer pre-cooked/readymade food in my diet	48.9	27.5	13.1	6.4	4.2

**Table 4:** Exploratory factor analysis.

Items	Mean	SD	F 1	F 2	F 3	F 4
MCW—positive influence on memory	3.3	1.14	0.909			
MCW—positive influence on heart health	3.6	1.07	0.891			
MCW—positive influence on blood vessels	3.7	1.04	0.875			
MCW—positive influence on cholesterol levels	3.3	1.14	0.869			
MCW—positive influence on blood sugar	3.2	1.16	0.815			
MCW—positive influence on weight	2.9	1.24	0.786			
MCW—positive influence on energy level	3.0	1.25	0.760			
MCW—positive influence on metabolism	3.2	1.16	0.712			
MCW—positive influence on neurovegetative diseases	3.3	1.16	0.635			
Pays attention to fat intake	3.1	1.12		0.856		
Pays attention to sugar intake	3.4	1.09		0.826		
Pays attention to fibre intake	3.1	0.99		0.782		
Pays attention to calorie intake	2.9	1.14		0.778		
Pays attention to salt intake	3.2	1.09		0.759		
Pays attention to vitamin intake	3.3	1.06		0.734		
Pays attention to additive intake	3.7	1.08		0.612		
Being health-conscious	3.6	0.95		0.607		
Label: Lower risk of heart disease	3.5	1.26			0.989	
Label: Reduces cholesterol levels	3.7	1.24			0.978	
Label: Positive influence on health	3.7	1.20			0.633	
Label: Reduces vascular disease	3.5	1.20			0.603	
Label: Contains antioxidative components	3.8	1.22			0.406	
Label: Do not consume with medicine	4.2	1.21				0.860
Label: Do not drink and drive	4.4	1.19				0.813
Label: Not for younger than 18	4.2	1.08				0.728
Label: Consume in moderate amounts	4.1	1.17				0.705
Label: Do not consume during pregnancy and breastfeeding	3.9	1.24				0.600
Eigenvalues			7.993	3.233	3.423	2.597
% variance			29.604	11.973	12.678	9.620
% cumulative variance			29.604	41.577	54.255	63.874
Cronbach’s α			0.946	0.909	0.917	0.853

**Table 5:** Internal correlation coefficient of test scale.

Variable	Measurement	Correlation Coefficient
Moderate wine consumption (MCW)	MCW—positive influence on memory	0.860 ***
	MCW—positive influence on heart health	0.849 ***
	MCW—positive influence on blood vessels	0.847 ***
	MCW—positive influence on cholesterol levels	0.883 ***
	MCW—positive influence on blood sugar	0.865 ***
	MCW—positive influence on weight	0.846 ***
	MCW—positive influence on energy level	0.829 ***
	MCW—positive influence on metabolism	0.815 ***
	MCW—positive influence on neurovegetative diseases	0.871 ***
Health behaviour (HB)	Pays attention to fat intake	0.850 ***
	Pays attention to sugar intake	0.816 ***
	Pays attention to fibre intake	0.824 ***
	Pays attention to calorie intake	0.800 ***
	Pays attention to salt intake	0.807 ***
	Pays attention to vitamin intake	0.826 ***
	Pays attention to additive intake	0.716 ***
	Being health-conscious	0.691 ***
Label—health benefit claims (LHBC)	Label: Lower risk of heart disease	0.881 ***
	Label: Reduces cholesterol levels	0.917 ***
	Label: Positive influence on health	0.865 ***
	Label: Reduces vascular disease	0.911 ***
	Label: Contains antioxidative components	0.838 ***
Label—warnings (LW)	Label: Do not consume with medicine	0.827 ***
	Label: Do not drink and drive	0.809 ***
	Label: Not for younger than 18	0.819 ***
	Label: Consume in moderate amounts	0.775 ***
	Label: Do not consume during pregnancy and breastfeeding	0.742 ***

Note: *** significant at α = 0.001.

**Table 6:** The descriptive statistics of the constructs.

Variable	Mean	SD	Correlation			
			MCW	HB	LW	LHBC
MCW	3.4	0.95	1	0.189 ***	−0.001	0.612 ***
HB	3.4	0.81		1	0.127 *	0.306 ***
LW	3.7	1.06			1	0.280 ***
LHBC	4.2	0.89				1

Note: * significant at α = 0.05. *** significant at α = 0.001.

**Table 7:** Results of the regression analysis.

Variable	Model 1	Model 2	Model 3
Constant	2.888	2.448	2.018
Age	0.096	0.034	−0.046
Gender	−0.173 **	−0.196 ***	−0.134 **
Education	0.094	0.056	0.049
Income	0.007	0.006	−0.025
Profession	0.000	0.014	0.005
HB		0.199 ***	0.039
LHBC			−0.173 ***
LW			0.651 ***
R^2^	0.045	0.080	0.428
Adjusted R^2^	0.031	0.064	0.414
F	3.153 **	4.838 ***	30.862 ***
R^2^ Change	0.045 **	0.035 ***	0.348 ***
F Change	3.153	12.708	100.256

Note: ** significant at α = 0.01. *** significant at α = 0.001.

## Data Availability

The original contributions presented in this study are included in the article. Further inquiries can be directed to the corresponding author.

## Appendix A

**Table A1:** Dimensions of the perception of health benefits perception of associated with moderate wine consumption and dietary habits and non-obligatory wine label information.

Variables	Description	Author	Year
MCW—positive influence on memory	Better cognitive tests for wine consumption Red wine as protective for women	[15] [16]	2011 2018
MCW—positive influence on heart health	Med, Dash diet, and MWC of red wine as protective factors Resveratrol and heart failure Effects on cardiovascular diseases	[50] [7]	2017 2017
MCW—positive influence on blood vessels	Resveratrol and vascular inflammation Effects on cardiovascular diseases	[50] [7]	2017 2015
MCW—positive influence on cholesterol levels	Resveratrol and oxidative stress Resveratrol treatments	[50]	2017
MCW—positive influence on blood sugar	Med, Dash diet, and MWC of red wine as protective factors Limitation of sugar in diet	[12]	2015
MCW—positive influence on weight	Flavonoid polyphenols in metabolic diseases and inflammation Antioxidant properties of polyphenols have also been shown to be effective in metabolic diseases Effects on obesity	[50] [7]	2017 2017
MCW—positive influence on energy level	Functional foods make people more energetic	[51]	2020
MCW—positive influence on metabolism	Consuming polyphenols from fruit and red wine	[12]	2015
MCW—positive influence on neurovegetative diseases	Better cognitive tests for wine consumption Women, cognitive scores, impairment, decline Red wine as protective for women Red wine reduced the risk of AD in men	[15]. [13] [16]	2011 2005 1999
Pays attention to fat intake	Limitation of fats, e.g., saturated Non-saturated fat intake (olive oil)	[10]	1999
Pays attention to sugar intake	Limitation of sugar in diet, Med diet positive for diabetes	[12]	2015
Pays attention to fibre intake	Adding fibre into everyday diet, eating a balanced diet	[54]	2019
Pays attention to calorie intake	Food choice questionnaire Diet proposals and nutrient substitutions	[55] [52]	1998 2020
Pays attention to salt intake	Food choice questionnaire Reduction of sodium Sodium and hypertension	[55] [52]	1998 2020
Pays attention to vitamin intake	Eating a balanced diet Vitamins as food supplements	[54] [66]	2019 2014
Pays attention to additive intake	Other substances use Xylitol Eating food without additives	[52] [51]	2020 2020
Being health-conscious	Conscious choices of foods in diet, Mediterranean, DASH, and AHEI diets as choices for healthy life	[12] [53]	2015 2017
Label: Lower risk of heart disease	Health impact with eco/environmental label	[7]	2017
Label: Reduces cholesterol levels	Better willingness to pay for wine with health aspects	[3]	2006
Label: Positive influence on health	Health aspects and WTP	[56]	2008
Label: Reduces vascular disease	Wine and health Olive oil and heart health	[8] [67]	2016 2014
Label: Contains antioxidative components	Wine and health	[8]	2016
Label: Do not consume with medicine	Opinion on label—do not consume with medicine	[21]	2017
Label: Do not drink and drive	Opinion—after drinking do not drive	[21]	2017
Label: Not for younger than 18	Ban for children under 18, concern of overconsumption	[21] [58]	2017 1999
Label: Consume in moderate amounts	Trilateral survey highlighted what MWC is	[7] [58]	2017 1999
Label: Do not consume during pregnancy and breastfeeding	In France, health warning on labels is obligatory	[7]	2017
